# Cluster analysis and phylogenetic relationship in biomarker identification of type 2 diabetes and nephropathy

**DOI:** 10.4103/0973-3930.60003

**Published:** 2010

**Authors:** Satya Vani Guttula, Allam Appa Rao, G. R. Sridhar, M. S. Chakravarthy, Kunjum Nageshwararo, Paturi V. Rao

**Affiliations:** Department of Marine Living Resources, Andhra University, India; 1 Department of College of Engineering, Andhra University, India; 2 Department of Endocrine and Diabetes Centre, Nizams Institute of Medical Sciences, Hyderabad, Andhra Pradesh, India; 3 Department of Endocrinology, Nizams Institute of Medical Sciences, Hyderabad, Andhra Pradesh, India

**Keywords:** Cluster analysis, phylogenetic relation, microarray, type 2 diabetes and nephropathy

## Abstract

Cluster analysis of DNA microarray data that uses statistical algorithms to arrange the genes according to similarity in patterns of gene expression and the output displayed graphically is described in this article. Hierarchical clustering is a multivariate tool often used in phylogenetics, comparative genomics to relate the evolution of species. The patterns seen in microarray expression data can be interpreted as indications of the status of the genes responsible for nephropathy in peripheral blow cells of type 2 diabetes (T2DN). Out of 415 genes totally expressed in the 3 DNA chips it was concluded that only 116 genes expressed in T2DN and in that only 50 are functional genes. These 50 functional genes are responsible for diabetic nephropathy; of these 50, some of the genes which are more expressed and responsible are AGXT: Alanine-glyoxylate aminotransferase, RHOD: Ras homolog gene family, CAPN6: Calpain 6, EFNB2: Ephrin-B2, ANXA7: Annexin A7, PEG10: Paternally expressed 10, DPP4: Dipeptidyl-peptidase 4 (CD26, adenosine deaminase complexing protein 2), ENSA: Endosulfine alpha, IGFBP2: Insulin-like growth factor binding protein 2, 36kDa, CENPB: Centromere protein B, 80kDa, MLL3: Myeloid/lymphoid or mixed-lineage leukemia 3, BDNF: Brain-derived neurotrophic factor, EIF4A2: Eukaryotic translation initiation factor 4A, isoform 2, PPP2R1A: Protein phosphatase 2 (formerly 2A), regulatory subunit A, alpha isoform. Fifty genes and their nucleotide sequences are taken from NCBI and a phylogenetic tree is constructed using CLUSTAL W and the distances are closer to each other concluding that based on the sequence similarity and evolution the genes are expressed similarly. Literature survey is done for each gene in OMIM and the genes responsible for diabetic nephropathy are listed.

## Background

Nephropathy (T2DN) is a frequent complication of diabetes mellitus. Renal failure in diabetes is mediated by multiple pathways. The risk factors for progression of chronic kidney disease (CKD) in type 2 diabetes Mellitus (DM) have not been fully elucidated. Although uncontrolled blood pressure (BP) is known to be deleterious, other factors may become more important once BP is treated. Asian Indians with type 2 diabetes mellitus (T2D) have higher susceptibility to diabetic nephropathy (T2DN), the leading cause of end stage renal disease and morbidity in diabetes. Peripheral blood cells play an important role in diabetes, yet very little is known about the molecular mechanisms of PBCs regulated in insulin homeostasis. In this study, the global gene expression changes in PBCs in diabetes and diabetic nephropathy to identify the potential candidate genes, expression and their phylogenetic relationship according to the different clusters in diabetes and nephropathy. We utilized the data of gene expression values from our earlier publication.[1]

### Microarrays

High throughput techniques are becoming more and more important in many areas of basic and applied biomedical research. Microarray techniques using cDNAs are much high throughput approaches for large scale gene expression analysis and enable the investigation of mechanisms of fundamental processes and the molecular basis of disease on a genomic scale. Several clustering techniques have been used to analyze the microarray data.

As gene chips become more routine in basic research, it is important for biologists to understand the biostatistical methods used to analyze these data so that they can better interpret the biological meaning of the results. Strategies for analyzing gene chip data can be broadly grouped into two categories: Discrimination and clustering.

Discrimination requires that the data consist of two components. The first is the gene expression measurements from the chips run on a set of samples. The second component is data characterizing. For this method, the goal is to use a mathematical model to predict a sample characteristic, from the expression values. There are a large number of statistical and computational approaches for discrimination ranging from classical statistical linear discriminate analysis to modern machine learning approaches such as support vector machines and artificial neural networks.

In clustering, the data consist only of the gene expression values. The analytical goal is to find clusters of samples or clusters of genes such that observations within a cluster are more similar to each other than they are to observations in different clusters. Cluster analysis can be viewed as a data reduction method in that the observations in a cluster can be represented by an ‘average’ of the observations in that cluster. There are a large number of statistical and computational approaches available for clustering. These include hierarchical clustering and k-means clustering from the statistical literature and self-organizing maps and artificial neural networks from the machine learning literature. While these algorithms are relatively equivalent in terms of performance, the focus of this paper will be on hierarchical clustering.[2]

## Materials and Methods

Microarray data of gene expression values from Paturi V Rao's paper Gene expression profiles of peripheral blood cells in type 2 diabetes and nephropathy in Asian Indians is taken. Data analyzed here were collected on spotted DNA microarrays, The additional Data File1 contains 416 genes which are expressed in 3 different DNA microarray samples that is T2D vs. C, T2DN vs. C, T2DN vs. T2D. These 416 Genes with their expression values are given to Cluster 3.0 tool and a dendrogram is generated.

### Hierarchical clustering

Several different algorithms will produce a hierarchical clustering from a pair-wise distance matrix. Cluster analysis is often used to bring similar individuals into groups. In hierarchical clustering, individuals are successively integrated based on the dissimilarity matrix computed by data, to obtain a dendrogram which contains inclusive clusters. In the context of microarray analysis, it is used to classify unknown genes or cases of disease. Several different algorithms will produce a hierarchical clustering from a pair-wise distance matrix. The algorithms begin with each gene by itself in a separate cluster. These clusters correspond to the tips of the clustering tree (dendrogram). The algorithms search the distance matrix for the pair of genes that have the smallest distance between them and merge these two genes into a cluster. Many algorithms follow this series of steps to produce hierarchical clustering of data. We will consider an average linkage algorithm. Average linkage is one of many hierarchical clustering algorithms that operate by iteratively merging the genes or gene clusters with the smallest distance between them followed by an updating of the distance matrix.

### Heat maps

Hierarchical clustering is used to produce what have been called ‘heat maps’ in papers reporting on microarray data analyses. The heat map presents a grid of colored points where each color represents this case, the three columns represent samples and the rows represent 416 genes. In the heat map colors at a particular point (i.e., row by column coordinate) are assigned to represent the level of expression for that gene (row) in the sample (column) with red corresponding to high expression, green corresponding to low expression and black corresponding to an intermediate level of expression. The ordering of the rows and columns was determined using hierarchical clustering and the associated dendrogram for the samples shown. In this example, 3 samples were clustered. The heat map gives an overall view of the 416 genes expression levels.[3]

When 416 genes were given to the Cluster 3.0 tool, genes got divided into four clusters. We have selected the fourth cluster as these genes are highly expressed with red color in T2DN. There are 116 listed genes and they are displayed in Additional data File2 with functions. Out of 116 Genes, only 50 are functional, this data is stored in additional Datafile3.

## Results

**Figure 1:**
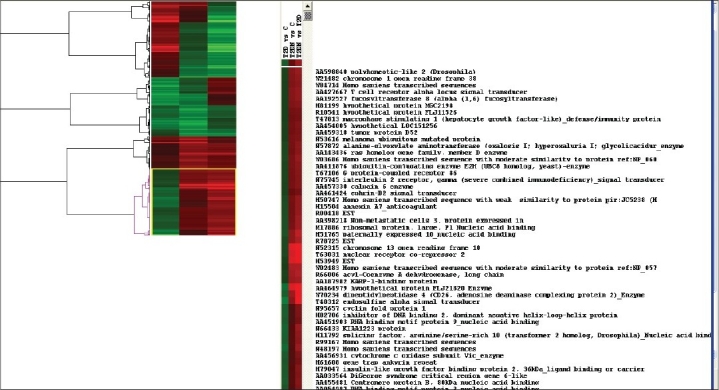
Cluster Tree View Result – The List of genes expressed in type 2 diabetic nephropathy

Gene expression profiling of mRNA from PBCs from six diabetics with nephropathy (T2DN), six diabetics without nephropathy (T2D) and six non-diabetic subjects (C), using 13,824 human sequence verified cDNA clones revealed significant differential expression of 416 genes.[1] Hierarchical clustering of significant genes revealed distinct gene expression signatures for diabetes and diabetic nephropathy. A Phylogenetic relationship between the gene clusters is shown with distances and a cladogram is constructed [Figure 1].

Now the 50 functional genes are taken along with their sequences and a phylogenetic tree is constructed using CLUSTAL W- A multiple sequence alignment tool Available on internet.[5] A phylogenetic Tree with the distances is displayed in the picture 2 and the distances can be seen from Additional Data file 4.

Finally, each functional gene is taken and the OMIM database is searched for its role in humans. All the 50 genes are related closely as the distances between them is closer so their rate of expression is also similar. In the OMIM database the genes were keenly studied and identified some of the genes which are more responsible for diabetic nephropathy are identified; such as AGXT: Alanine-glyoxylate aminotransferase, RHOD: Ras homolog gene family, CAPN6: Calpain 6, EFNB2: Ephrin-B2, ANXA7: Annexin A7, PEG10: Paternally expressed 10, DPP4: Dipeptidyl-peptidase 4 (CD26, adenosine deaminase complexing protein 2), ENSA: Endosulfine alpha, IGFBP2: Insulin-like growth factor binding protein 2, 36kDa, CENPB: Centromere protein B, 80kDa, MLL3: Myeloid/lymphoid or mixed-lineage leukemia 3, BDNF: Brain-derived neurotrophic factor, EIF4A2: Eukaryotic translation initiation factor 4A, isoform 2, PPP2R1A: Protein phosphatase 2 (formerly 2A), regulatory subunit A, alpha isoform.

## Discussion

We have focused on presenting an overview of hierarchical clustering of microarray data, emphasizing the relationship between a dendrogram and spatial representations of genes. We believe this relationship provides an intuitive understanding of how to analyze microarray data and can make it easier to interpret the results of a cluster analysis in a biological framework. The fact that the ‘heat maps’ found in most of the microarray publications are based on hierarchical clustering indicates that an understanding of this general method is valuable to those who are just beginning to read the microarray literature and even to those who are using supervised methods. We have used cluster analysis software, which is available online in Eisen laboratories and the version is Cluster3.0.

Identification of candidate genes in peripheral blood could provide easily accessible biomarkers to monitor diabetic nephropathy and these are AGXT: Alanine-glyoxylate aminotransferase,[6] RHOD: Ras homolog gene family,[7] CAPN6: Calpain 6,[8] EFNB2: Ephrin-B2,[9,10] ANXA7: Annexin A7,[11] PEG10: Paternally expressed 10,[12] DPP4: Dipeptidyl-peptidase 4 (CD26, adenosine deaminase complexing protein 2),[13] ENSA: Endosulfine alpha,[14] IGFBP2: Insulin-like growth factor binding protein 2, 36kDa,[15] CENPB: Centromere protein B, 80kDa,[16] MLL3: Myeloid/lymphoid or mixed-lineage leukemia 3,[17] BDNF: Brain-derived neurotrophic factor,[18] EIF4A2: Eukaryotic translation initiation factor 4A, isoform 2,[19] PPP2R1A: Protein phosphatase 2 (formerly 2A), regulatory subunit A, alpha isoform.[20] Phylogenetic relationship shows that with similar expression values there is an evolutionary relation shown by the phylogenetic tree.[4]

**Figure 2:**
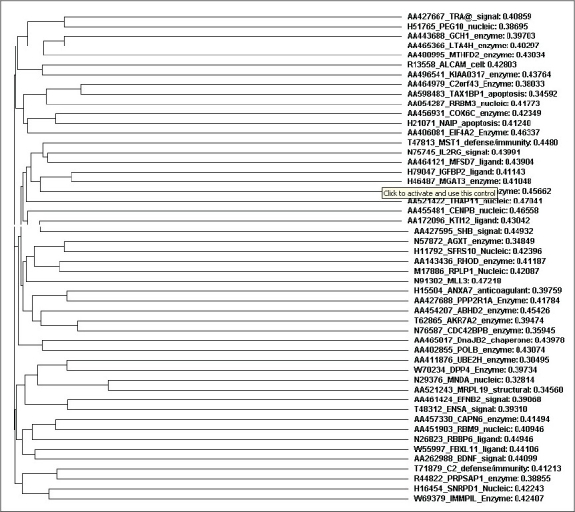
Clustal W- Phylogenetic tree of the 50 genes
